# Repeated Low-Level Blast Exposure Alters Urinary and Serum Metabolites

**DOI:** 10.3390/metabo13050638

**Published:** 2023-05-08

**Authors:** Austin Sigler, Jiandong Wu, Annalise Pfaff, Olajide Adetunji, Paul Nam, Donald James, Casey Burton, Honglan Shi

**Affiliations:** 1 Department of Chemistry, Missouri University of Science and Technology, Rolla, MO 65409, USA; 2 Phelps Health, Rolla, MO 65401, USA

**Keywords:** blast exposure, homovanillic acid, biomarkers

## Abstract

Repeated exposure to low-level blast overpressures can produce biological changes and clinical sequelae that resemble mild traumatic brain injury (TBI). While recent efforts have revealed several protein biomarkers for axonal injury during repetitive blast exposure, this study aims to explore potential small molecule biomarkers of brain injury during repeated blast exposure. This study evaluated a panel of ten small molecule metabolites involved in neurotransmission, oxidative stress, and energy metabolism in the urine and serum of military personnel (*n* = 27) conducting breacher training with repeated exposure to low-level blasts. The metabolites were analyzed using HPLC—tandem mass spectrometry, and the Wilcoxon signed-rank test was used for statistical analysis to compare the levels of pre-blast and post-blast exposures. Urinary levels of homovanillic acid (*p* < 0.0001), linoleic acid (*p* = 0.0030), glutamate (*p* = 0.0027), and serum *N*-acetylaspartic acid (*p* = 0.0006) were found to be significantly altered following repeated blast exposure. Homovanillic acid concentration decreased continuously with subsequent repeat exposure. These results suggest that repeated low-level blast exposures can produce measurable changes in urine and serum metabolites that may aid in identifying individuals at increased risk of sustaining a TBI. Larger clinical studies are needed to extend the generalizability of these findings.

## 1. Introduction

Military personnel encounter repeated low-level blast exposures in the course of training and combat operations [1,2]. Such repeated exposure can produce biological and clinical changes that resemble mild traumatic brain injury (TBI) [3], as evidenced by clinical symptomology [4], neuroimaging [5,6], and long-term neurological deficits [7,8]. In the short term, blast-related TBI can further increase the risk of repeat injury upon subsequent exposures [5,9]. As a result, suspected blast-related TBIs need to be assessed for medical intervention in a timely manner to mitigate long-term health consequences.

Current approaches to evaluating blast-related TBIs, including neuropsychological evaluations, acute injury surveillance, and imaging studies, suffer from subjectivity, insensitivity to mild injuries, and poor applicability in the field that lead to delayed diagnosis and inadequate treatment [10]. Recent efforts to develop novel molecular biomarkers have already revealed several altered proteins, including neuron-specific enolase, S100B, and glial fibrillary acidic protein, in serum and cerebrospinal fluid [11,12,13,14,15,16,17]. However, the complex cascade of pathophysiological changes that accompany blast exposure, including ionic flux, indiscriminate neurotransmitter release, increased glycolysis, and oxidative stress, present an unexplored source of potential metabolic biomarkers [18,19,20,21,22,23].

To this end, we recently developed a highly sensitive high-performance liquid chromatography-tandem mass spectrometry (HPLC-MS/MS) method to enable measurement of a panel of urinary metabolites related to several distinct pathological processes involved in brain injury, including homovanillic acid (HVA), glutamic acid (Glu), lactic acid (Lac), linoleic acid (LA), arachidonic acid (AA), pyruvic acid (Pyr), methionine sulfoxide (MetSO), *N*-acetylaspartic acid (NAA), 5-hydroxyindoleacetic acid (5-HIAA), and F2α-isoprostane (F_2α_) [19]. In the current study, we set out to determine whether specific metabolites in our panel, whether alone or in combination, can produce measurable changes in response to repeated low-level blast exposures commonly encountered by military personnel and law enforcement agencies in training environments.

## 2. Materials and Methods

### 2.1. Study Design and Participants

This study was conducted at the Urban Mobility Breacher Course at Fort Leonard Wood, Missouri, USA, where military personnel receive comprehensive training on how to gain entry into buildings using explosive techniques. The training program consists of a two-week training course with the first week comprising classroom-based learning and the second week comprising field exercises involving repeated blast exposures. This course structure enabled a longitudinal study design in which baseline data collected in the first week prior to blast exposure could be compared with blast-related data collected during the second week on an individualized basis to better control for external factors. The research protocol and informed consent process for the study were approved by the Phelps Health Institutional Review Board (IRB), University of Missouri IRB, and the Human Research Protection Program of the Army Research Laboratory.

A total of 27 active military personnel (25 males and two females, age range: 20–35) were recruited to participate in this study from the Urban Mobility Breacher Course at Fort Leonard Wood, MO, USA in June 2021. All participants encountered the same blast exposure comprising a variety of light charges (0.03, 0.07, 0.11, and 0.15 lbs C4) and heavy charges (10.44 lbs C4) daily over the course of several days. Such low-level blast exposure has been demonstrated to cause behavioral and structural abnormalities in mice models [24]. During the field exercises, trainees formed a line starting at approximately 50 ft behind the explosive charge. Trainees then sequentially moved in position through the line with subsequent blasts to evenly distribute cumulative blast exposure throughout the duration of the field exercises.

### 2.2. Materials

All chemicals and solvents used in this study were high purity (>97% or better). Chemical standards for HVA, Glu, Lac, Pyr, MetSO, NAA, AA, LA, isotopically-labeled internal standards glutamic acid-^13^C_5_,^15^N (Glu-^13^C_5_,^15^N), 5-hydroxyindoleacetic acid 4,6,7 D_3_-acetic-3-D_2_ (5-HIAA-D_5_), and *N*-acetyl-*L*-aspartic acid-2,3,3-D_3_ (NAA-D_3_) were purchased from Sigma-Aldrich, (St. Louis, MO, USA). In addition, 5-HIAA, F_2α_, LC-MS-grade methanol, isopropanol, acetonitrile (ACN), and optima™ grade formic acid (FA) were purchased from Fischer Scientific (Hampton, NH, USA). Isotopically labeled internal standard F_2α_-isoprostane-D_4_ (F_2α_-D_4_) was purchased from Cayman Chemicals (Ann Arbor, MI, USA). Ultrapure water (18.2 MΩ·cm) was generated in-house by a Millipore Elix-3 purification system (Millipore, Billerica, MA, USA).

### 2.3. Sample Collection

During the first week prior to blast exposure, three daily spot urine specimens consisting of first or second morning voids were collected from each participant. Additionally, a 5 mL blood specimen was obtained on the first day of sample collection. During the second week involving blast exposure, three additional daily spot urine specimens consisting of first or second morning voids were collected after the blast exposures of each day. Urine specimens were collected approximately 16 h after the previous day’s blast exposure. Finally, a 5 mL blood specimen was collected on the final day of sample collection.

Blood specimens were spun down after 30 min clotting time to collect serum aliquots that were frozen immediately at −80 °C until analysis. The urine samples were frozen immediately at −80 °C on the collection site and transferred to the Missouri University of Science and Technology for analysis. Urine specimens were subsequently thawed, aliquoted, and refrozen at −80 °C until analysis, except for one urine aliquot per specimen to determine urine specific gravity.

### 2.4. Sample Preparation

Samples were prepared according to published methods [19,25] with slight modification to include additional internal standards for enhanced accuracy and sensitivity. Briefly, after specimens were thawed and equilibrated to room temperature, each 100 µL aliquot was added to a 900 µL mixture of 800 µL ACN and 100 µL ultrapure water containing a 10 mg/L mixture of internal standards (5-HIAA-D_5_, Glu-^13^C_5_,^15^N, NAA-D_3_, and F_2α_-D_4_) in 1.5 mL microcentrifuge tubes. The samples were vortexed and diluted prior to HPLC-MS/MS analysis according to our published method [19].

### 2.5. HPLC-MS/MS Analysis

Each sample was analyzed using two previously described HPLC-MS/MS methods to quantify the ten metabolites in this study using a Shimadzu UFLC system coupled to a 4000 QTRAP tandem mass spectrometer (AB SCIEX, Concord, ON, Canada) [19,25]. The first HPLC-MS/MS method that analyzed the eight metabolites was the same as our previously published method [19] with a further improvement of method robustness by including additional isotope-labelled internal standards. The second method for quantifying LA and AA was adapted from a published method [25]. The method was slightly modified to reduce the HPLC-MS/MS analysis time. Briefly, an Ascentis Express C18 column (10 cm × 2.1 mm, 2.7 µm particle size, Phenomenex, Torrance, CA, USA) was used. The analytes were eluted with a flow rate set to 0.3 mL/min under a gradient elution program with eluent A (3:2 ultrapure water:ACN with 10 mM ammonium acetate and 0.1% formic acid) and eluent B (9:1 isopropanol:ACN with 10 mM ammonium acetate and 0.1% FA). The gradient began with 20% B, followed by a linear increase over 3 min to 100% B, which was maintained for 2.5 min before decreasing to 20% B over 0.5 min and equilibrated at 20% B for 10 min before the next injection. Optimized ion source conditions included: ion source temperature of 400 °C, ion spray voltage of −4500 V, curtain gas pressure at 15 psi, ion source gas 1 pressure at 50 psi, and ion source gas 2 pressure at 30 psi. The mass transitions and optimized other conditions are listed in Table 1. Both HPLC-MS/MS methods were validated to ensure performance in urine and serum samples before being applied to analyses of field blast samples.

### 2.6. Urine Specific Gravity Measurements

Urinary metabolite concentrations were normalized to urine concentration-dilution to account for an individual’s hydration status and time since last urination using urine specific gravity (USG) [26]. USG was measured refractometrically with a Reichert digital Clinic-Chek Digital Handheld Refractometer after urine specimens were equilibrated to room temperature.

### 2.7. Quality Assurance and Quality Control

All analytical methods were validated prior to sample analysis. These include analyses of blanks, calibration standards, method detection limits, reproducibility of replicate samples, and sample matrix spikes. During sample analysis, one or more blanks, sample duplicates, and sample spike recovery checks were performed for each sample batch to ensure acceptable method performance and data quality. In addition, samples with metabolite concentration above the calibration range were diluted and reanalyzed.

### 2.8. Data Processing and Statistical Analyses

USG-adjusted analyte concentrations were expressed in terms of μg/L. Analyte concentrations below the limit of detection (LOD) were treated as zero for statistical analyses. The LOD was determined as signal to noise ratio equal to 10 using matrix-matched blanks for both serum and urine specimens. Statistical analyses were conducted using GraphPad Prism 9 (San Diego, CA, USA). Nonparametric Wilcoxon signed-rank tests were performed using untransformed analyte levels from paired groups (individualized comparisons of urine and serum specimens before and after blast exposure). Significance levels for Wilcoxon matched-pairs signed-rank tests were set at 0.05 (two-tailed).

## 3. Results

### 3.1. Method Performance

Both HPLC-MS/MS methods performed acceptably well in urine and serum samples in terms of accuracy and precision. For Method 1, the relative standard deviations (RSD) for the eight urinary metabolites ranged from 2.7% to 6.7%, and spiked recoveries ranged from 81.4% to 111.8%. The RSDs for the eight serum metabolites ranged from 2.4% to 9.4%, and spiked recoveries ranged from 86.1% to 121.4%. For analysis of AA and LA (Method 2), the RSDs ranged from 1.6 to 4.2% in urine and from 3.4 to 4.2% in serum. The spiked recoveries ranged from 94.1% to 106.0% for urine and 103.0% to 108.7% for serum. The method quantification detection limit for LA was 10 µg/L in both urine and serum. The method quantification detection limits for AA were 1 µg/L and 10 µg/L in urine and serum, respectively.

### 3.2. Effect of Blast Exposure on Urinary Metabolites

**Figure 1:**
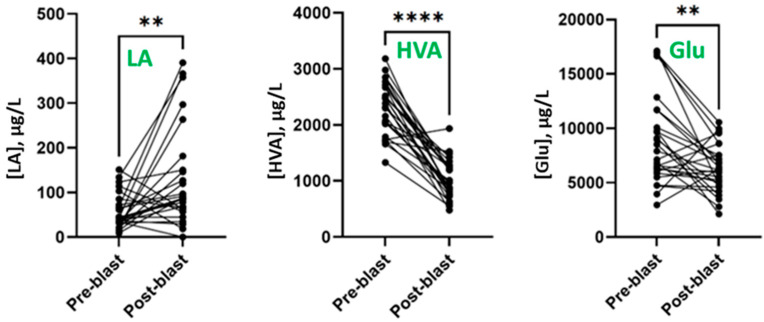
Pre- and post-blast LA, HVA, and Glu levels in urine for each participant (*n* = 27). Black circles represent three-day average, and lines connect pre- and post-blast levels for each participant. ** *p* < 0.01, **** *p* < 0.0001 determined by Wilcoxon matched pairs signed-rank test.

Statistical comparisons using the average pre-blast and post-blast metabolite concentrations for each participant were performed to examine the effects of blast exposures on urinary metabolite levels. Wilcoxon signed-rank comparisons revealed that significant changes occurred in urinary levels of LA, HVA, and Glu following blast exposure (Figure 1).

Post-blast concentrations of urinary HVA were significantly lower than pre-blast levels (pre-blast median of 2385 μg/L versus post-blast median of 969.4 μg/L, *p* < 0.0001). Urinary Glu levels also decreased significantly following blast exposure (pre-blast median of 7906 μg/L versus post-blast median of 6161 μg/L, *p* = 0.0027), though the difference was not as dramatic as seen in HVA. In contrast, urinary LA levels significantly increased after blast exposure (pre-blast median of 42.7 μg/L, versus post-blast median of 173.9 μg/L, *p* = 0.0030). Wilcoxon signed-rank analyses for other analytes in the panel were also performed; however, no significant changes between the pre- and post-blast samples were observed.

**Figure 2:**
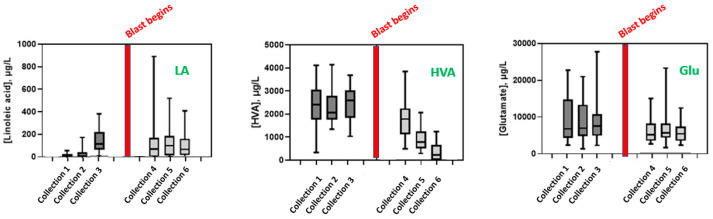
Box-and-whisker plots illustrating urinary LA, HVA, and Glu levels throughout the study. Boxes represent the interquartile range, and horizontal bars represent the median for each day.

In addition to aggregated comparisons before and after blast exposure, changes in urinary metabolites throughout the training program were assessed to identify trends in response to blast exposure. This approach revealed that urinary HVA continues to decrease with repeated blast exposure (Figure 2). However, significant temporal trends were not observed in urinary Glu and LA.

The summary statistics, including medians and ranges, for all urinary metabolites before and after blast exposure have been provided in Table 2. It should be noted that urinary F_2α_ was not meaningfully detected in this sample cohort and was excluded from data analysis. Pre-concentration methods may be employed in future studies to better detect this analyte, although it was not employed in the current study owing to practical limitations. In addition, the limited sample size and cumulative blast exposure may have limited our ability to discern measurable changes in the other metabolites following blast exposure.

### 3.3. Effect of Blast Exposure on Serum Metabolites

**Figure 3:**
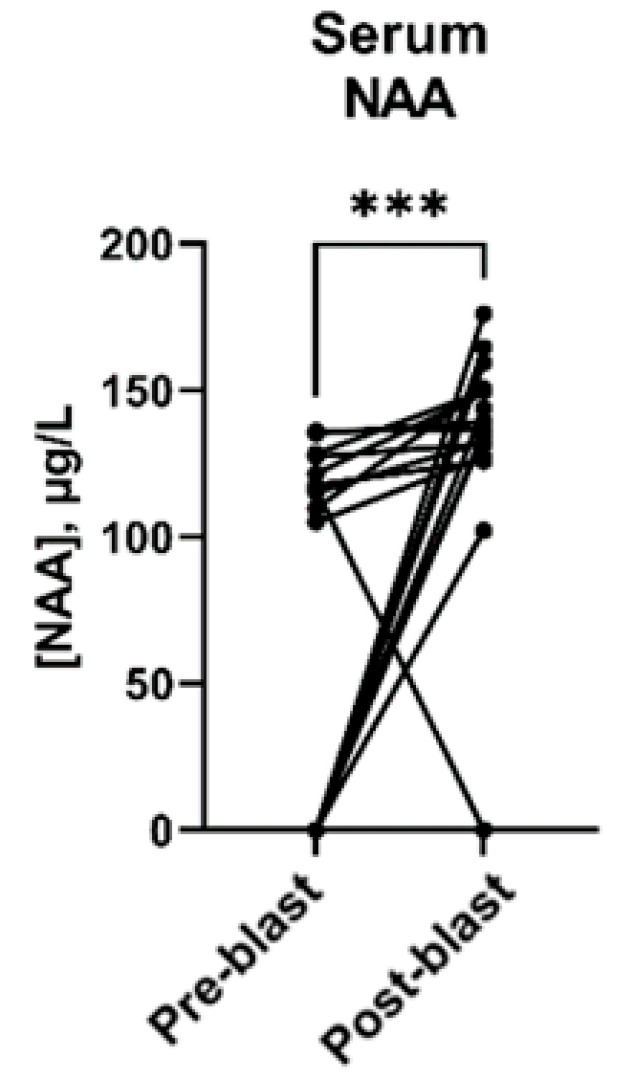
Serum NAA levels before and after blast exposure for each participant (*n* = 17). Black circles represent three-day average, and lines connect pre- and post-blast levels for each participant. *** *p* < 0.01, determined by Wilcoxon matched pairs signed-rank test.

Wilcoxon signed-rank analyses were similarly performed for serum metabolites. These comparisons revealed that serum NAA levels increased significantly following blast exposure (pre-blast median of 107.3 μg/L versus a post-blast median of 136.5 μg/L, *p* = 0.0006) (Figure 3). The concentrations of all serum metabolites tested have been summarized in Table 3.

The presentation of different metabolites as well as differing metabolite concentrations in urine and serum may be attributed to the unique temporal profile of each metabolite following blast exposure. Notably, the biomarker kinetics of these selected metabolites have not yet been established and further study is warranted. In addition, urine samples were collected more frequently than serum samples in this study, providing enhanced temporal resolution of the underlying biomarker kinetics. Furthermore, the relatively poor detection of serum fatty acids in this study was likely an adverse result of the protein precipitation step during sample preparation. Serum fatty acids are found disproportionately bound to lipoproteins in the bloodstream, causing protein precipitation methods, such as the one used in the current study, to inadvertently remove fatty acids from the sample [27].

## 4. Discussion

Urinary HVA decreased significantly and continuously with repeated blast exposure. As the primary metabolite of dopamine, reductions in urinary HVA following blast exposure may indicate altered neurotransmission [20] and dopamine formation in damaged neurons [28]. Dopamine systems including HVA have been widely implicated in cognitive deficits following TBI. For example, reduced dopamine transmission has been previously reported in plasma samples collected from rodent models using controlled cortical impact and fluid percussion techniques to induce traumatic brain injuries [29]. Decreased HVA has also been reported in human cerebrospinal fluid (CSF) after head injury [30,31]. Interestingly, CSF levels of 5-HIAA have similarly been found to be unchanged following head injuries despite decreased HVA [30,31]. This previous work corroborates our finding of decreased HVA and unchanged 5-HIAA in urine following blast exposure. Notably, previous work using the same breaching training program noted elevated levels of several serum proteins related to axonal injury [17]. In addition, recent clinical work has demonstrated decreased levels of urinary HVA decreases in individuals with clinically confirmed TBI up to six months following injury with concentrations being correlated to injury severity [32]. Similarly, HVA concentrations in CSF have been shown to be more pronounced in patients with a longer duration of unconsciousness [30]. Taken together, these findings, corroborated by the current study, would suggest that HVA may be a useful biomarker for brain injury, including blast-related brain injury, to improve characterization of injury severity and recovery [33]. In particular, the current study suggests that urinary HVA, which may be more readily collected and analyzed, may hold clinical value.

Urinary Glu was also observed to decrease following repeated low-level blast exposure, although not to the same extent as seen with urinary HVA. This comparison suggests that the response of urinary Glu may not be as sensitive to repeated low-level blasts as urinary HVA. In addition, altered metabolism of Glu to glutamine, which was not evaluated in the current study, may have impacted urinary levels of Glu following blast exposure. Nevertheless, decreased levels of urinary Glu, a primary neurotransmitter, further suggest altered neurotransmission following repeated low-level blast exposure. Previous microdialysis studies in human and rodent models have shown an immediate rise in extracellular glutamate levels following blunt-force TBI while magnetic resonance spectroscopy (MRS) studies have shown a decrease in total glutamate in human brains under similar insults [34]. Moreover, Glu imbalances are different for acute, subacute, and chronic TBI and the underlying metabolic processes responsible for these observed differences remain poorly understood [34]. To the best of our knowledge, the current study is the first to assess whether urinary Glu can produce measurable changes in response to repeated low-level blast exposure, and further work is needed to determine the generalizability of this finding.

LA has been widely reported to increase in the brain following brain injury [35,36,37]. In the current study, urinary LA was similarly found to increase following repeated low-level blast exposure. While the metabolic role of LA in blast-related injury is not well established, fatty acid oxidation associated with injury-related oxidative stress and altered neurotransmission have previously been suggested [35]. In addition, it should be noted that a large variance in urinary LA was observed among study participants, suggesting significant variability in dietary sources, as LA is the most consumed polyunsaturated fatty acid in the US diet [38]. Participant diets were not recorded in the current study, and future studies assessing the role of urinary LA in TBI should consider controlling for dietary factors.

Serum NAA also showed significant changes following blast exposure. As a highly abundant metabolite in brain tissue, decreased levels of NAA have been reported in the brain following blunt-force brain injury using MRS, suggesting potential leakage into the bloodstream [39]. The increased serum levels observed in the current study provide supporting evidence of NAA leakage into the bloodstream following brain injury. While serum levels have not previously been reported in the context of traumatic brain injury, increased serum levels of NAA have been reported in patients with amyotrophic laceral sclerosis characterized by the progressive degeneration of nerve cells [40]. Regional reduction of NAA has also been used as a marker of neuronal or axonal loss in several neurological disorders and even TBI [40]. NAA derivatives have additionally been implicated in altered glutamatergic transmission [41]. The combined changes seen for urinary glutamate and serum NAA provide further evidence that repeated blast exposure may contribute to alterations in neurotransmission and the metabolites that normally regulate those processes.

## 5. Conclusions

This study evaluated a panel of ten urinary and serum metabolites following repeated low-level blast exposure in military personnel during explosive breaching exercises. Several metabolites in this panel were found to have statistically significant changes during and after blast exposure compared to individualized baseline values. Specifically, urinary levels of HVA, Glu, and LA were significantly altered following repeated low-level blast exposure. Furthermore, the presentation of different metabolites and their reported changes following blast exposure in urine compared to serum suggested a dynamic temporal profile that may be explored in future studies to determine biomarker kinetics. In this way, the current study demonstrated that repeated low-level blast exposure, at levels experienced in training courses regularly conducted by military personnel and law enforcement agencies, can produce measurable changes in serum and urine metabolites. With additional study, these blast-sensitive metabolites may offer clinicians with new screening tools to assess and characterize blast-related brain injuries.

## Figures and Tables

**Table 1:** Optimized parameters for Method 2. The upper ion pairs were used for quantification while the lower ion pairs were used for confirmation.

Compound	Ion Pairs	Declustering Potential (DP, V)	Collision Energy (CE, V)	Collision Cell Exit Potential (CXP, V)
Arachidonic Acid (AA)	349.4/303.6	−35	−18	−7
	349.4/259.6	−35	−24	−17
Linoleic Acid (LA)	325.4/279.6	−30	−18	−7
	325.4/45.2	−30	−30	−1

**Table 2:** Pre- and post-blast levels (μg/L) of dilution-corrected urinary metabolites (*n* = 27). Statistically significant *p*-values have been bolded.

Analyte	Pre-Blast		Post-Blast		*p*-Value
	Median	Range	Median	Range	
Linoleic acid (LA)	42.7	<LOD–352	173.9	<LOD–857	**0.0030**
Homovanillic acid (HVA)	2320	300–4108	864.6	<LOD–3850	**<0.0001**
Lactic acid (Lac)	933.7	65.18–2466	897.1	120.4–2724	0.7140
*N*-Acetylaspartic acid (NAA)	5831	2784–9879	6638	3127–12,926	0.2687
Arachidonic acid (AA)	18.82	6.045–59.14	26.87	8.483–81.41	0.2792
Pyruvic acid (Pyr)	1461	553.4–3731	1397	349.8–1959	0.2792
Methionine sulfoxide (MetSO)	105.2	11.91–398.6	138.6	61.54–302.3	0.1855
5-Hydroxyindoleacetic acid (5-HIAA)	2776	1149–5515	2397	936.5–5157	0.9153
Glutamic acid (Glu)	7906	1927–27,132	6161	2122–24,891	**0.0027**

**Table 3:** Pre- and post-blast dilution-corrected levels (μg/L) of serum metabolites (*n* = 18). Statistically significant *p*-values have been bolded.

Analyte	Pre-Blast		Post-Blast		*p*-Value
	Median	Range	Median	Range	
Linoleic acid (LA)	<LOD	<LOD–9.154	<LOD	<LOD–34.69	>0.999
Homovanillic acid (HVA)	<LOD	<LOD–513.7	<LOD	<LOD–382.3	0.4121
Lactic acid (Lac)	146,806	78,478–419,083	128,171	9011–373,531	0.1964
*N*-Acetylaspartic acid (NAA)	107.3	<LOD–136.0	136.5	<LOD–176.1	**0.0006**
Arachidonic acid (AA)	<LOD	<LOD–7.913	<LOD	<LOD–29.36	0.8203
Pyruvic acid (Pyr)	9003	2858–19,318	11,221	<LOD–26,216	0.2288
Methionine sulfoxide (MetSO)	<LOD	<LOD–175.4	<LOD	<LOD–141.4	0.9219
5-Hydroxyindoleacetic acid (5-HIAA)	<LOD	<LOD–338.2	<LOD	<LOD	0.0625
Glutamic acid (Glu)	2954	1118–4330	2827	<LOD–5108	>0.999

## Data Availability

All data presented in this manuscript are available on request from the corresponding authors.
